# Research Progress on the Role of Zinc Finger Protein in Colorectal Cancer

**DOI:** 10.1002/cnr2.70123

**Published:** 2025-03-14

**Authors:** Tang Yu, Jiumei Zhao, Ziwei Li, Chenglong Pan, Jialing Liu, Kepu Zheng, Xiaohao Wang, Yan Zhang

**Affiliations:** ^1^ The Third Affiliated Hospital of Kunming Medical University Kunming Medical University Kunming China; ^2^ Chongqing Nanchuan District People's Hospital Chongqing Medical University Chongqing China; ^3^ Department of Gynecology and Obstetrics, Chongqing Health Center for Women and Children Women and Children's Hospital of Chongqing Medical University Chongqing China; ^4^ Department of Infectious Diseases, Key Laboratory of Molecular Biology for Infectious Diseases (Ministry of Education), Institute for Viral Hepatitis, The Second Affiliated Hospital Chongqing Medical University Chongqing China

**Keywords:** colorectal cancer, mechanism of action, signaling pathway, treatment, zinc finger protein

## Abstract

**Background:**

Colorectal cancer is one of the most prevalent malignancies worldwide, with a tendency of increasing incidence in developed countries, which poses a significant threat to the patients' physical and mental health.

**Recent Findings:**

The process of gene transcription affects the important physiological functions of cells, so the normal expression of transcription factors is an important prerequisite for maintaining cellular homeostasis. Changes in the level of zinc finger proteins, the most prevalent transcription factor, may play an important trigger for the development of colorectal cancer. Different zinc finger proteins play different roles in terms of promoting or inhibiting cancer development.

**Conclusion:**

This paper briefly reviews the classification, functional characteristics, and expression changes of zinc finger proteins in colorectal cancer, it focuses on how they regulate gene transcription, influence on common signaling pathways, and their potential for translational studies and clinical applications. The objective is to stimulate new ideas for their study of colorectal cancer while also providing foundational information to guide drug development and treatment strategies for colorectal cancer patients in clinical settings.

## 1 Introduction

In recent years, the prevalence of various types of malignancies has gradually increased due to changes in people's lifestyles and dietary habits. Among them, colorectal cancer (CRC) stands out as one of the most common cancers worldwide and the third most common factor associated with death in cancer patients [1]. CRC occurs mainly in the mucosal epithelium of the colon and rectum and is a prevalent malignancy. According to epidemiological studies, CRC has the highest incidence and the third highest mortality rate in China, with a 5‐year survival rate of approximately 32% [2]. However, the precise pathogenesis of this tumor still requires further research, with genetics, environment, nutrition, and accelerated aging likely to be key factors (Table 1).

**TABLE 1:** Zinc finger protein structural domains and the substrates they bind.

Binding substrates	Zinc finger domain
DNA	C2H2, KRAB
Methylated DNA	C2H2, FYVE
RNA	C2H2, CHHC
Lipid	C2H2, FYVE
Protein	C2H2, RING, ZZ‐Type
Ubiquitin	KRAB, PBZ, UBP
SUMO	MYM‐Type
Modified histones	PHD, C5HC2
Poly(ADP‐ribose)	PBZ

Patients with CRC are difficult to distinguish from hemorrhoids due to the lack of characteristic symptoms in the early stages and the presence of hemorrhoid‐like blood in the stool [3]. It is well‐known that tumorigenesis and progression depend on regulatory pathological changes, such as cell invasion, migration, extracellular matrix degradation, and tumor angiogenesis [3], and that there are many molecular‐level alterations in the development of these pathological changes. Therefore, studying the molecular mechanisms underlying CRC development in order to identify more sensitive biomarkers and new therapeutic targets to improve the early diagnosis and detection of CRC has become a key focus and a significant challenge in clinical trials.

**FIGURE 1:**
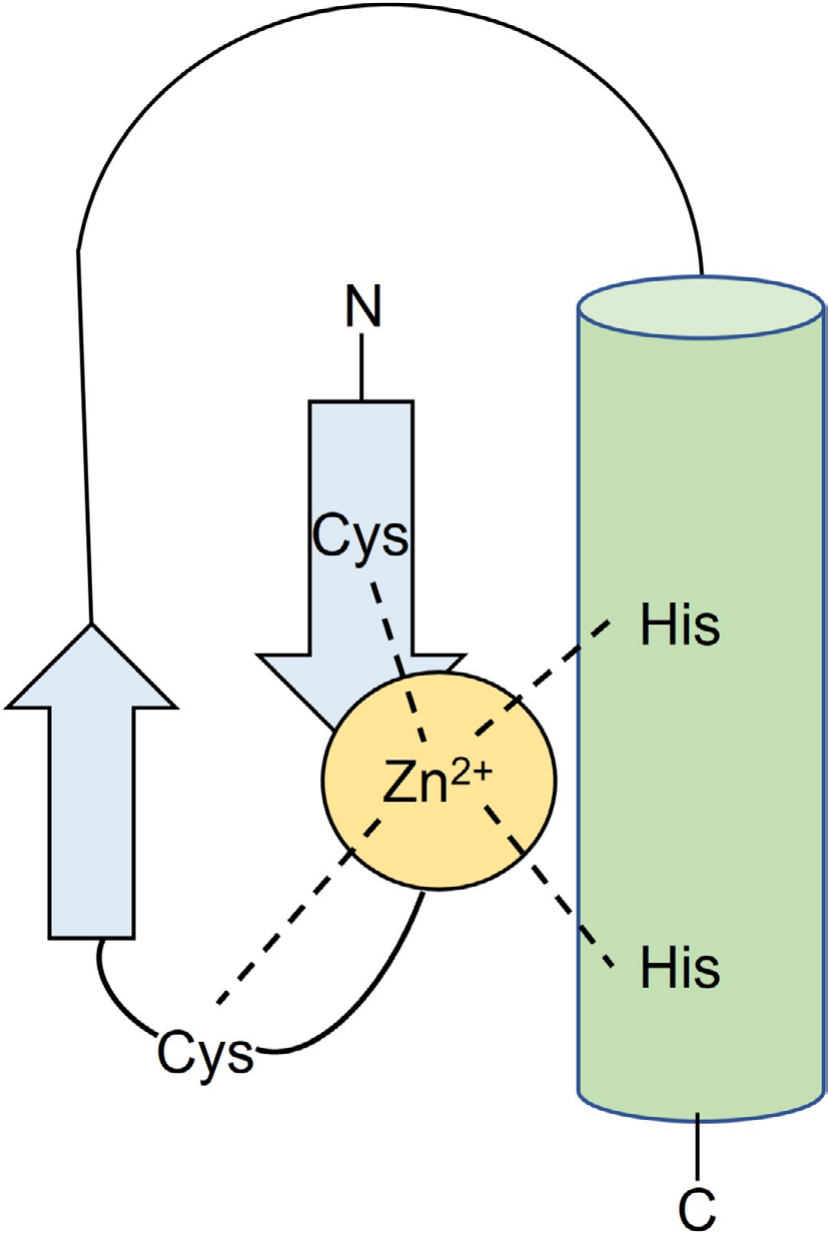
Schematic representation of the major structures of zinc finger proteins [4]. There is a pair of cysteine residues at the N‐terminus of the zinc finger and a pair of histidine residues at the C‐terminus. These four residues form a cavity in space that accommodates exactly one Zn ion. Because Zn ions can stabilize the α‐helix structure in the model, the α‐helix can be embedded in the major groove of DNA. Therefore, all proteins containing zinc fingers can bind to DNA or RNA. His: Histidine residues, Cys: Cysteine residues, Zn^2+^: Zn ion.

Zinc finger proteins (ZNFs), named after their “finger‐like” structures, consist of cysteine (Cys) and histidine (His) bound to Zn^2+^, which is β‐folded and α‐helical to form a stable tetrahedral structure [4] (Figure 1). Studies have shown that it can bind to DNA, RNA, proteins, and lipids, and they act as important transcription factors that regulate gene expression [5, 6]. ZNFs play a crucial role in various biological processes, including transcriptional regulation, post‐transcriptional modification, ubiquitin‐mediated protein degradation, actin targeting, signal transduction, cell migration, and DNA repair. They are also involved in cell differentiation, regulation of gene expression, embryonic development, and several other important phenomena [7]. ZNFs play different roles in different tumor types, and even within different subtypes of the same tumor due to their diverse molecular regulatory mechanisms that can either promote or inhibit tumor development. In this paper, we provide a brief review of the classification and functional characteristics of ZNFs. We discuss the changes in the expression levels of specific ZNFs in CRC and their regulation of gene transcription. Furthermore, we explore their effects on common signaling pathways and present clinical translation studies, with a view to providing new ideas for their study in CRC and providing basic information for the use of drugs or treatment for CRC patients in clinical settings.

## 2 ZNFs Classification and Its Functional Characteristics

**FIGURE 2:**
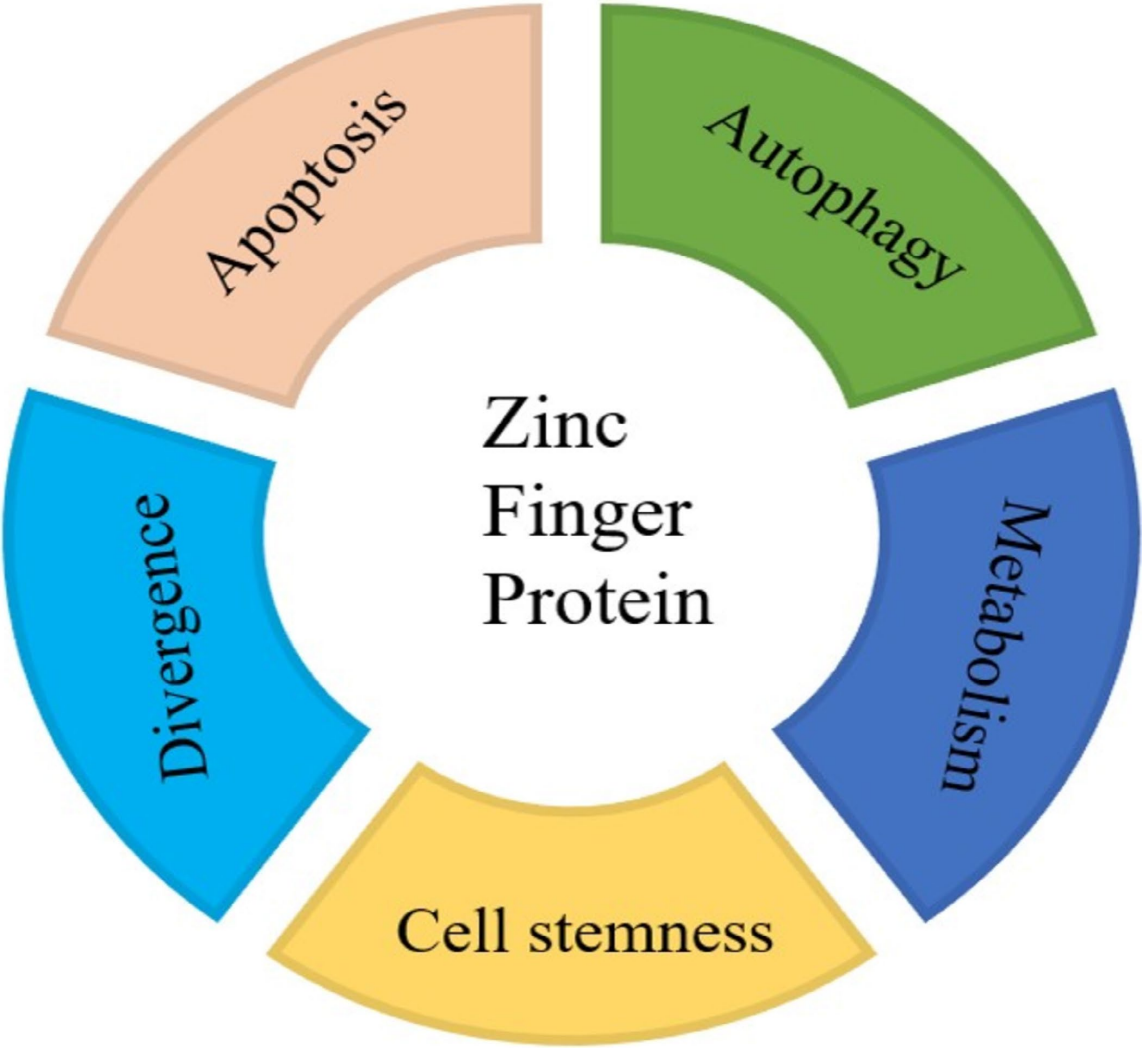
Zinc finger protein has major influence on biological functions in the cell [10]. Zinc finger proteins are involved in a variety of important cellular physiological activities, including cell stemness, divergence, apoptosis, autophagy, and metabolism.

ZNFs are the most commonly expressed class of proteins in mammals, with approximately 1% of the human genome sequence encoding ZNFs. Their structures have been classified into various types based on the combination of conserved Cys and His residues bound to Zn^2+^. Examples of these structural types included C2H2, C2HC, C3H, C4, C3HC4, C4HC3, C6, C8, and various combined types [8]. The C2H2 structure is the largest subgroup of all ZNFs, accounting for approximately 2% of the human genome, and the C2H2 ZNFs family has more than 5000 members [9]. ZNFs contain multiple unique structural domains with diverse characteristics such as binding targets, binding modes, and affinities. These are able to recruit and bind to a variety of molecules, diversifying the function of ZNFs while enhancing gene stability [10]. The combination of different zinc finger structures allows ZNFs to have multiple gene regulatory functions that are associated with different cellular states and stimuli, including apoptosis, autophagy, cell differentiation, human metabolism, and stem cell maintenance [10] (Figure 2).

**FIGURE 3:**
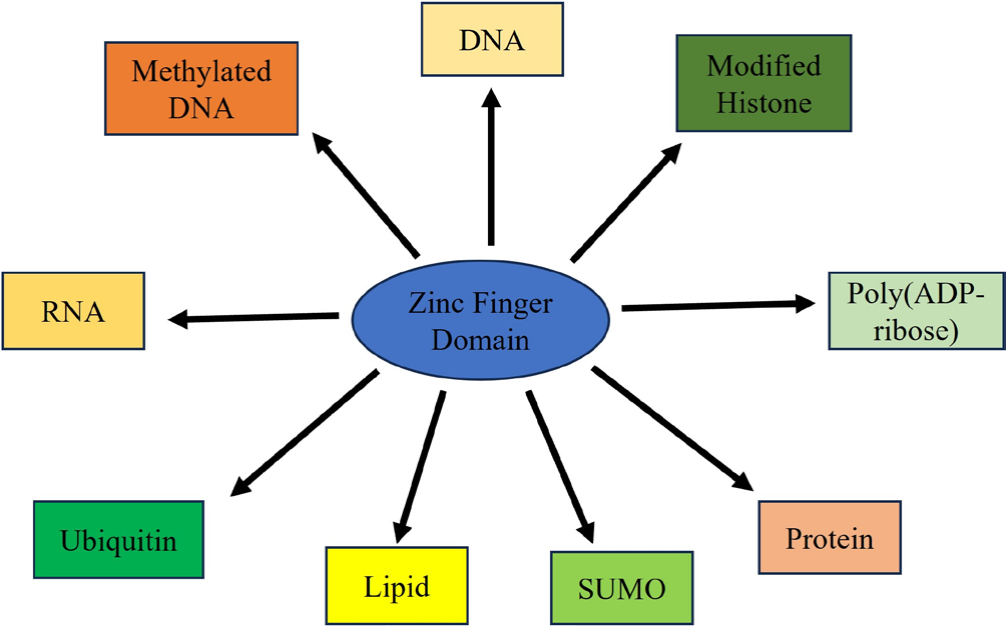
Zinc finger protein binding substrate [11]. Zinc finger proteins contain many domains that can bind to their substrate, including certain proteins or nucleic acids. Such as SUMO, lipid, ubiquitin, RNA, methylated DNA, DNA, modified histone, poly(ADP‐ribose), and protein.

ZNFs can participate in the regulation of downstream target gene transcription through diverse functional structural domains in conjunction with transregulatory progenitors. Specifically, ZNFs can regulate the transcription of downstream genes by interacting with the transcriptional activation domain (TAD), transcriptional inhibitory domain (TID), transcription factors, co‐activators, co‐repressors, other proteins, DNA methylation, histone modifications, and other chromosomal modifiers [11] (Figure 3). Thus, ZNFs play a very critical role in gene regulation.

## 3 Changes in the Expression Abundance of ZNFs in CRC

In recent years, the ZNFs family has been proven to mediate the occurrence and development of CRC by regulating the transcription and expression of related genes, which is an important marker in assisting tumor diagnosis and prognosis [12, 13].

Among the specific members of the ZNF family, the ZNF217 gene is localized at 20q13.2. It has been reported that ZNF217 expression is significantly higher in CRC cancer tissues than in paraneoplastic tissues and that low expression of ZNF217 in CRC cells may inhibit cell migration and invasiveness [14].

ZNF281 (also known as ZNP99 or ZBP99), is a Krüppel‐type zinc finger transcriptional regulator located on chromosome 1q32.1 [15]. It is a key regulator of embryonic stem cell (ESC) differentiation and tissue development [16]. Recent studies have shown that high expression of ZNF281 in CRC cells promotes invasive and migratory capacities and is strongly associated with poor prognosis in CRC patients [17, 18]. These findings indicate that ZNF281 may play an important role in the development and progression of CRC.

ZNF139, another member of the Krüppel family, is located on chromosome 7q21.3‐q22.1 and acts as a transcription‐associated protein [19]. ZNF139 has been found to be highly expressed in CRC tissues and is correlated with differentiation, infiltration, TNM staging, and lymph node metastasis. These observations suggest a close linkage between the ZNF139 gene and the emergence, development, and malignancy of CRC [19].

ZNF148 (also known as ZBP89, ZBP‐89, or ZFP148), a multifunctional zinc finger transcription factor involved in the regulation of cell proliferation and death, is highly expressed in tumor cells [20]. However, one study found that reduced expression of ZNF148 in CRC is strongly associated with several adverse clinical features, including lymph node metastasis, advanced TNM stage, poor differentiation, high recurrence rates, short overall survival, and disease‐free survival [21]. This observation may be attributed to the presence of two selectively spliced isoforms of ZNF148, namely ZNF148FL and ZNF148ΔN [22]. Functional antagonism between ZNF148FL and ZNF148ΔN has been observed, where increased expression of ZNF148FL decreases the expression of ZNF148ΔN, thus promoting proliferation, migration, and invasion of human CRC cells. Conversely, increased expression of ZNF148ΔN decreases the expression of ZNF148FL, thereby promoting apoptosis and inhibiting cell proliferation, migration, and invasion in human CRC cells [22].

ZNF384 is highly expressed in CRC and it can affect the metastatic and proliferative capacity of CRC cells through upregulation of hypoxia‐inducible factor 1 alpha (HIF1a) and matrix metalloproteinase 2 (MMP2) [23].

ZFP57, the ESC‐specific transcription factor, also is a ZNF expressed in ESCs, It has been shown to play an important role in tumor formation. Shoji et al. [24] have shown that it is highly expressed in CRC, which they verified through in vitro and in vivo experiments and clinical samples, showing that high expression of ZFP57 is highly correlated with liver metastasis in CRC. In addition to the ZNFs mentioned above, there are a number of ZNFs that also show altered expression in CRC. Most are upregulated in expression in CRC, such as ZNF545 [25]; ZNF746 which promotes the progression of CRC by upregulating c‐Myc [26]; and ZNF3 which promotes the proliferation and migration of CRC by upregulating MMP1 [27]. ZNF726 is also associated with a shorter overall survival in CRC patients, etc. Conversely, reduced expression of ZNFs, including ZNF750, ZNF671, and ZNF516 [27, 28, 29], has also been observed in CRC, and their reduced expression suggests a poorer prognosis for CRC patients.

In summary, ZNFs may act as a double‐edged sword, as their increased or decreased expression can be associated with the development of CRC (Table 2). Additionally, different isoforms of the same ZNF may have distinct roles in CRC. Therefore, understanding the expression patterns of ZNFs in CRC may provide valuable prognostic information for CRC patients.

**TABLE 2:** Changes in the expression abundance of common ZNFs in CRC.

Name of zinc finger protein	Expression level tumor vs. normal	References
ZNF217	Up	[14]
ZNF281	Up	[16, 17, 18]
ZNF139	Up	[19]
ZNF384	Up	[23]
ZNF545	Up	[24]
ZNF746	Up	[25]
ZNF3	Up	[26]
ZNF148	Down	[20, 21, 22]
ZNF750	Down	[27]
ZNF671	Down	[28]
ZNF516	Down	[29]

## 4 Effects of ZNFs on Transcriptional Regulation of Genes in CRC

Metastasis‐associated genes 2 and 3 (MTA2 and MTA3), which belong to the MTA family and are components of the nucleosome remodeling deacetylase (NuRD) complex, play direct or indirect roles in transcriptional regulation [30]. MTA1 has been found to upregulate the expression of Snai1 and Slug, while silencing MTA1 reduces the binding of Snai1 and Slug to the E‐cadherin promoter, resulting in decreased E‐cadherin expression in CRC cells [31]. Prostate cancer‐associated transcript 1 (PCAT1) an oncogenic long non‐coding RNA (lncRNA) located on chromosome 8, has been implicated in the development of various cancers, including CRC [32]. Based on bioinformatics, Zhang et al. [33] identified ZNF217 as a potential target of lncRNA PCAT1. Their results suggest that lncRNA PCAT1 may promote CRC progression by coordinating ZNF217 to regulate the MTA2/MTA3/Snai1/E‐cadherin pathway involved in epithelial‐mesenchymal transition (EMT).

One of the frequently mutated genes in CRC is the tumor suppressor gene p53, commonly known as the guardian of the genome [34]. p53 is activated in response to various stress signals, such as DNA damage or oncogene activation, and orchestrates diverse downstream responses, including DNA repair, cell cycle arrest, senescence, metabolism, and cell death [35]. Acting primarily as a transcription factor, p53 controls the expression of hundreds of target genes [36]. Mutations in p53 not only disrupt the transactivation of typical p53 target genes but may also confer novel oncogenic properties and promote tumorigenesis [37]. In colorectal cells, ZNF575 can directly bind to the promoter sequence of TP53, which in turn promotes the expression of p53 protein and inhibits the proliferation of CRC cells [38].

Taken together, these studies have shown that ZNFs have transcription factor activity in CRC, and have different transcriptional activities. Some ZNFs can promote the expression of oncogenes and some can promote the expression of tumor suppressor genes, thereby affecting the occurrence and development of CRC. Therefore, ZNFs can be used as a detection index for the occurrence and development of CRC, or as a potential target for CRC treatment. Recent studies have indicated that ZNFs exhibit interactions with various proteins, influencing the transcriptional activity of genes independent of their function as transcription factors. ZNF280A to D are also members of the ZNF protein family, and their sequences are highly homologous, but the terminal C2H2 carboxyl terminal composition is different. ZNF280A [39, 40] and ZNF280B [41, 42] have been implicated in promoting tumorigenic by controlling the transcription of downstream target genes, while whether ZNF280C and ZNF280D are involved in tumorigenic roles is unclear. Recently, Ying et al. [43] found that ZNF280C plays a critical role in maintaining the level of H3K27me3 at the genomic spacer. ZNF280C counteracts the activity of CTCF and cohesion at these sites by recruiting the transcriptional repressor SMCHD1, which facilitates the retention of DNA CpG methylation and/or repressive histone marks, including H3K27me3. This process attenuates the local accessibility of CTCF binding, ultimately leading to gene silencing.

Collectively, in CRC, ZNFs can act as transcription factors to influence the expression of downstream genes, thereby exerting progenic or oncogenic effects. Moreover, they can also act as intermediary proteins, mediating interactions with other proteins to modulate the transcriptional regulation of genes. Consequently, ZNFs play an important role in the development of CRC.

## 5 Effects of ZNFs on Metastasis‐Related Pathways in CRC

CRC is physiologically characterized by the ability to undergo distant metastasis and its aggressive nature [44, 45]. Several studies have shown that ZNFs play a crucial role in the metastasis of CRC. The wingless‐type MMTV integration site family (WNT) signaling pathway, which includes both classical and non‐classical signaling pathways, is extensively studied, with the WNTt/β‐catenin signaling pathway being a classical and well‐known pathway. This pathway is highly conserved and regulates invasion, metastasis, apoptosis, and development in a various tumor cell, including CRC cells [46, 47]. Clinical studies have associated aberrant WNTt/β‐ catenin signaling with oncogene activation and frequent observations in CRC patients [48]. Studies have shown that ZNF281, ZNF276, and ZNF334 can promote the proliferation and metastasis of CRC cells by upregulating the WNTt/β‐catenin signaling pathway [49, 50, 51].

The JAK‐STAT pathway, a major mechanism in malignant hematological diseases, has also been reported to be highly activated in solid tumors, including CRC [51]. Hao et al. [52] discovered that ZNF460 is highly expressed in CRC patients and is associated with distal metastasis. This effect may be mediated through the activation of the JAK‐STAT pathway.

The MMPs signaling pathway is involved in cancer invasion and metastasis. Among MMPs, MMP2, known as gelatinase A, plays a key role in malignant cell migration by degrading type IV collagen. MMP2 is a potential prognostic biomarker, with elevated expression in cancer cells associated with poor survival outcomes in CRC [53]. Tang et al. [54] found that high ZNF384 expression promotes MMP2 upregulation, thereby promoting metastasis in CRC cells.

NANOG, a transcription factor for homologous chromosomes, plays a crucial role in regulating the self‐renewal of ESCs and has the ability to suppress differentiation [55]. Some tumor cells also have stemness, and the nanog gene is involved in the regulation of stemness of tumor cells [56]. The increased stemness of tumor cells can promote the metastatic ability of cancer cells [57]. Recent studies have shown that ZFP57 is positively correlated with nanong expression and appears to be positively correlated with liver metastasis in CRC [24]. The results suggest that ZNF can affect the metastatic ability of tumor cells by regulating the stemness of tumor cells. The process of EMT is a common phenomenon in the metastasis of tumor cells, and its metastatic ability seems to be related to the EMT process in CRC [58]. ZNF326 is a zinc finger protein that promotes tumor cell metastasis in CRC by affecting the EMT process [59].

**FIGURE 4:**
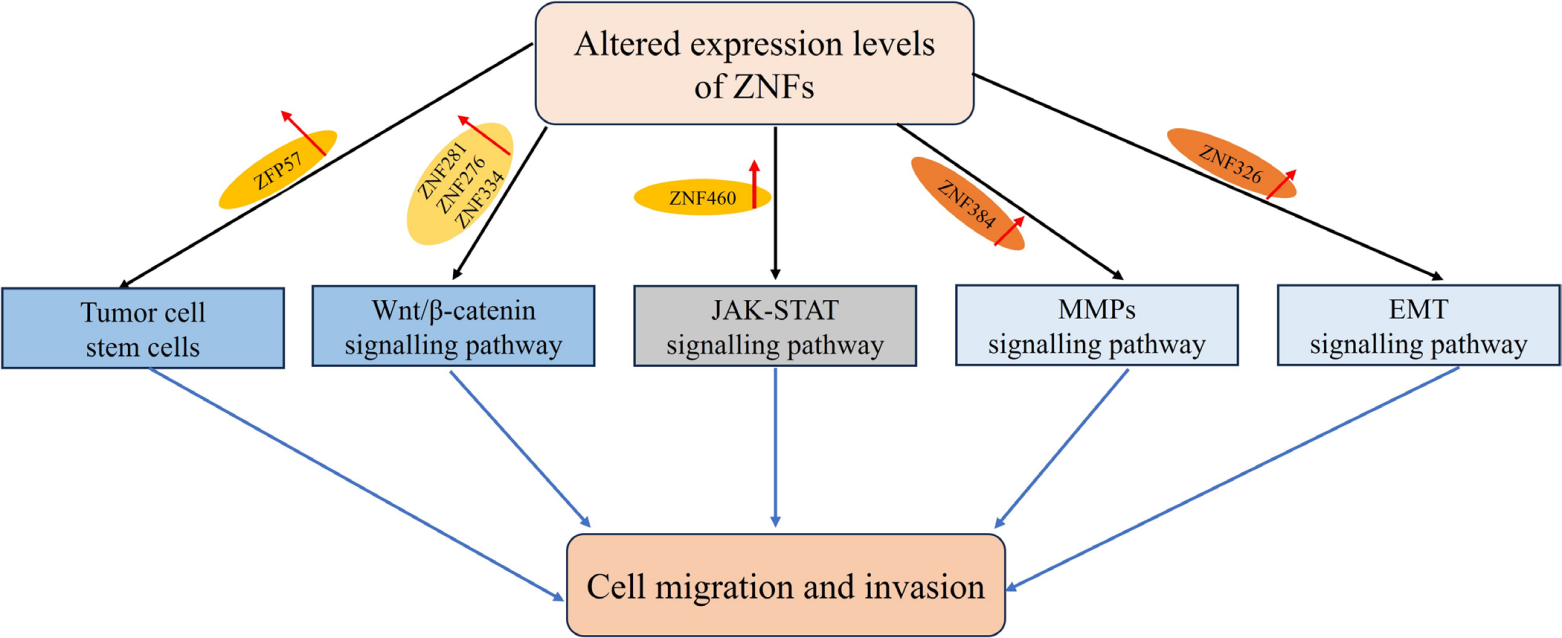
Effect of ZNFs on CRC metastasis‐related pathways [46, 47, 51, 52, 55]. Zinc finger protein plays an important role in the occurrence and development of CRC, and promotes the metastasis and invasion of CRC by activating some tumor‐related signaling pathways. For example, increased expression of ZFP57 activates tumor stemness signaling; ZNF281, ZNF276, and ZNF334 could activate WNT/ β‐catenin signaling pathway, ZNF460 could activate jak‐stat signaling pathway, ZNF384 could activate MMPs signaling pathway, ZNF326 could activate EMT signaling pathway. The activation of these pathways has an important link with the metastasis and invasion of CRC.

It can be seen that ZNFs mainly function as metastasis‐related factors (Figure 4), and they influence the migration of CRC cells by regulating signaling pathways such as WNT, JAK‐STAT, and MMPs. These findings highlight the strong association between ZNFs, distal metastasis, and the degree of malignant progression of CRC.

## 6 Possible Clinical Translation Study of ZNFs in CRC

PCNA (proliferating cell nuclear antigen), an indispensable nuclear protein in DNA synthesis, plays an important regulatory role in DNA replication, and its expression reflects the proliferative state of cells. Elevated PCNA expression is observed in various tumors including CRC and acts as a marker of tumor cell proliferation [60]. The survivin gene, a member of the inhibitor of apoptosis protein (IAP) family, is involved in CRC pathogenesis, and its inhibition can induce apoptosis in cancer cells [61]. Li et al. [62] demonstrated that inhibition of ZNF281 expression reduced PCNA and Survivin expression, and increased sensitivity to 5‐FU chemotherapy. This suggests that ZNF281 may regulate CRC cell proliferation and apoptosis by affecting the expression of PCNA and Survivin. Additionally, ZNF281 was found to be regulated by UCA1/miR‐ 23b‐3p signaling pathway in CRC cells. UCA1 promotes cellular autophagy and inhibit apoptosis through the miR‐23b‐3p/ZNF281 pathway, conferring CRC resistance to 5‐ FU [63]. This suggests that restoring the expression level of miR‐23b‐3p may be one of the effective strategies for CRC treatment. Thus, the UCA1/miR‐23b‐3p/ZNF281 signaling pathway may become a new target in CRC therapy. By inhibiting the expression of UCA1 and ZNF281 or increasing the expression of miR‐23b‐3p, it may be possible to improve the sensitivity of CRC cells to 5‐FU and thus improve the therapeutic effect.

ZNF331, a protein consisting of 463 amino acids, genetically located on chromosome 19q13.42, contains a KRAB structural domain and a zinc finger with 12 C2H2 [64]. ZNF331 acts as a tumor suppressor in CRC cells, inhibiting the proliferation of CRC cells and the growth of CRC cells in xenograft mice, indicating that ZNF331 is a potential tumor suppressor for CRC [65]. Moreover, ZNF331 is frequently methylated in CRC, its expression is regulated by promoter methylation, and ZNF331 methylation is a poor prognostic marker for CRC [66]. Similarly, the promoter of ZNF582 is frequently methylated and modified in CRC, showing an association with CRC metastasis [67]. These findings suggest that ZNFs are involved in the occurrence and development of CRC and may become a new target for CRC treatment and a molecular marker for prognostic assessment. Additionally, they provide new research ideas for the study of CRC caused by abnormal DNA methylation modification and offer a theoretical basis for the use of DNA demethylation drugs in clinical settings.

ZNF306, a member of the BTB/POZ subfamily in the ZNF family, is often regarded as a tumor suppressor and can bind to the promoter region of the CCND1 gene in CRC tissues, thereby inhibiting the expression of cytosolic Cyclin D1 [68]. In addition, ZNF306 can also target the methylation HIF1α promoter region in CRC tissues, resulting in the suppression of HIF1α expression [69]. It is known that tumor tissues increase the number of blood vessels in the tissues in order to overcome the hypoxic environment, providing a huge amount of oxygen to the tumor tissues. Elevated expression of HIF1A is often associated with the formation of tumor blood vessels, and the increase in neovascularization can contribute to the distal metastasis of tumor cells [70, 71, 72, 73]. Therefore, ZNF306 may serve as a marker for the development of distal metastasis in CRC, while anti‐angiogenic drugs or HFI1A antagonists can be used for CRCs caused by ZNF306 deficiency, thereby inhibiting distal metastasis in CRC.

JAK inhibitors, known for their ability to block the JAK‐STAT signaling pathway, have shown clinical efficacy in treating various immune diseases. In China, five JAK inhibitors have received approval for clinical use, including ruxolitinib, tofacitib, baricitinib, ubatinib, and abciatinib, for the treatment of atopic dermatitis, rheumatoid arthritis, inflammatory bowel disease, and myeloproliferative neoplasms [74]. So far, WNT pathway inhibitors are still under research status and have not yet entered clinical trials. Currently, the known WNT pathway inhibitors, including UBE2T, SM08502, and WNT‐C59, have demonstrated inhibitory effects on the migration ability of tumor cells [75, 76, 77]. As mentioned earlier, ZNFs affect the signaling pathway activities such as WNT, JAK‐STAT, etc. Therefore, the development and use of inhibitors of WNT and JAK‐STAT signaling pathways will probably also be effective clinical agents for the treatment of CRC patients with altered ZNF abundance. To think differently, if specific agonists or small‐molecule inhibitors targeting specific ZNFs are investigated and developed, is it possible to reverse the state of the WNT and JAK‐STAT pathways in CRC and thus influence the course of CRC development? There is a lack of research in this area. So further research in this area is warranted.

Immunotherapy is a common method to treat tumor [78]. Recent studies have shown that ZNFs may affect the immune response in tumor cells. Such as, ZNF721 inhibits T cell function and induces immune escape of tumor cells [79](8). The increased expression of ZNF580 seems to be associated with the validation response, increasing the difficulty of immunotherapy, and targeting ZNF580 seems to be a better means of immunotherapy [80]. Recent studies have also shown that ZNF4/7 seems to be related to the killing effect of T cells on tumor cells [81]. These results suggest that ZNFs may play an important role in tumor immunotherapy. It is well‐known that the prognosis and therapeutic response of CRC are associated with specific subtypes, and the common treatment modality is still surgical, with CRC lumpectomy as the mainstay, but there is often over‐treatment, postoperative complications, and the postoperative mortality rate is on the rise [82]. Fluorouracil, a uracil (FU)‐like drug, alone or in combination, has been the main clinical chemotherapeutic regimen for CRC, however, tumor resistance has severely limited its efficacy [71]. Although the international field has made great progress in drug‐targeted therapy and immunotherapy for CRC in recent years, the genetic status of KRAS, NRAS, BRAF, MSI/MMR, PIK3CA/PIK3CB, etc. in patients with CRC suggests that less than one‐third of patients can successfully benefit from drug‐targeted therapy and immunotherapy, and less than 8% of patients can benefit from PD‐1 [83, 84]. Therefore, the development and use of molecularly targeted drugs for CRC in the clinic remains a great challenge. If one wants to design corresponding targeted drugs for ZNFs to treat CRC, it is necessary not only to be clear about the effect of ZNFs on the development of broad‐spectrum CRC, but probably also to understand the expression of ZNFs in specific subtypes of CRC. However, so far, most studies have investigated ZNFs with broad‐spectrum CRC, and not many articles have reported the expression of ZNFs in specific subtypes of CRC, which suggests that the development of small‐molecule agonists or inhibitors of ZNFs may be used as broad‐spectrum drugs for CRC treatment. This needs to be supported by more experimental results.

## 7 Summary

The ZNF family, the largest gene family in the human body, plays a crucial role in cell differentiation, embryonic development, and the pathogenesis of various diseases. In particular, ZNFs play different roles in molecular regulatory mechanisms that promote or suppress tumor development in different tumor types or even different subtypes of the same tumor. Studies have shown that ZNFs also correlate with highly metastatic tumors such as breast cancer, ovarian cancer, and clear renal cell carcinoma, which seems to have some relevance to the CRC studies, (i.e., ZNFs seem to have a very strong correlation with tumor metastasis). Studies have shown that many ZNFs are closely associated with the development and progression of CRC, for example, ZNF217, ZNF281, ZNF139, ZNF148, ZNF384, and ZNF460. Their expression abundance in CRC increases and decreases, influencing the progression of CRC. Moreover, ZNFs can be divided into many of these subclasses, and are also able to act as transcription factors due to their structural characteristics, further contributing to the regulation of gene transcription. One of the reasons why CRC is a malignant tumor is that it is a highly metastatic and aggressive tumor with a generally poor prognosis for clinical patients. In CRC, it is most commonly associated with activation of the WNT signaling pathway, which is often associated with metastasis and invasion of tumors. Studies have reported that a variety of ZNFs are involved in the activation process of the WNT signaling pathway, and therefore ZNFs may serve as markers of CRC metastasis. At present, clinical drugs for the WNT signaling pathway have not been applied and are still at the stage of basic experiments. Revealing the regulatory mechanism of ZNFs on the WNT signaling pathway in CRC can provide a theoretical basis for the development of inhibitors of the WNT signaling pathway. CRC is highly aggressive and often has a poor prognosis. Immunotherapy has a good application prospect in tumors, but PD‐1 inhibitors have a poor response in CRC. Recent studies have found that ZNFs are also related to the activation of T cells, and the increased expression of some ZNFs seems to be related to the infiltration of T cells. For example, ZNF721, ZNF580, ZNF4/7, etc., can targeting these ZNFs increase the response to immunotherapy? As the largest gene family in the human body, ZNFs have a great impact on being used as therapeutic targets for CRC, but its role as a transcription factor or altered abundance of intermediary proteins causes changes in the expression of certain genes, and the downstream genes of these ZNFs contribute to the developmental process of CRC, and they may have more potential to become therapeutic targets, therefore, revealing the role of ZNFs and its mechanisms in CRC will be helpful to provide a basis for the development of molecularly targeted drugs for CRC.

## Author Contributions

X.W. and Y.Z. provided ideas for writing the review, J.Z., Y.T., Z.L. and J.L. wrote the first draft of this review, while C.P., Q.H., and K.Z. collected the information needed for the review, including references, pictures, and so on. At lastly, Dr. Y.T. revised the article.

## Disclosure

The authors have nothing to report.

## Ethics Statement

The authors have nothing to report.

## Consent

The authors have nothing to report.

## Conflicts of Interest

The authors declare no conflicts of interest.

## Data Availability

All references are from the pubmed public database with authentic and usable data.
